# Contrast-Enhanced MRI Is Useful for Prognostic Prediction on Idiopathic Bilateral Simultaneous Facial Nerve Palsy: A Case Report and Literature Review

**DOI:** 10.7759/cureus.54719

**Published:** 2024-02-22

**Authors:** Takato Shiozaki, Fumie Kaneko, Daichi Murakami, Masamitsu Kono, Muneki Hotomi

**Affiliations:** 1 Department of Otorhinolaryngology-Head and Neck Surgery, Wakayama Medical University, Wakayama, JPN

**Keywords:** facial nerve decompression, prognosis, signal intensity, contrast-enhanced mri, idiopathic bilateral simultaneous facial nerve palsy

## Abstract

Idiopathic bilateral facial nerve palsy is a rare condition and presents a diagnostic and prognostic challenge. Specifically, when bilateral nerves are damaged, it is difficult to predict the prognosis. We showcase the usefulness of contrast-enhanced magnetic resonance imaging (MRI) by providing information about localization and severity of degeneration of facial nerve. A 70-year-old Japanese man presented with bilateral simultaneous facial nerve palsy of House-Brackmann Grade VI on both sides. Contrast-enhanced MRI revealed bilateral intensity enhancement of intratemporal facial nerves. The signal intensity was higher on the left side than on the right side. Facial nerve decompression was performed on the left side. The left facial nerve palsy was finally improved eight months after the onset, while the right side was improved just under two months after the onset. Contrast-enhanced MRI for facial nerve palsy can provide valuable information for the evaluation of damaged facial nerves. In our patient’s case, it was useful as a prognostic predictor of bilateral facial nerve palsy.

## Introduction

Idiopathic facial nerve palsy frequently develops unilaterally, but rarely occurs bilaterally, with reports of between 0.3% and 2.0% of cases [1]. Bilateral idiopathic facial nerve palsy presents a diagnostic and prognostic challenge. A method that provides reliable prognostic information at an early stage of idiopathic facial nerve palsy is desired.

Electrophysiological tests such as nerve excitability test (NET) and electroneuronography (ENoG) are commonly used to predict the prognosis of facial palsy. However, when performed early, the results are usually inconclusive because it takes approximately one week to show Wallerian degeneration after the onset of symptoms [2]. Prediction of a poor or favorable outcome in the first days after onset is therefore difficult. Edema of the damaged facial nerve and subsequent degeneration and ischemia caused by compression are considered to be related to prognosis [3]. Corticosteroids are a commonly used treatment in the early stages of palsy [4]. Surgical decompression of a damaged facial nerve has been performed as a salvage treatment option [5].

Contrast-enhanced T1-weighted magnetic resonance imaging (MRI) is useful as a non-invasive imaging technique to assess facial nerve damage [6]. While MRI is deemed to have little prognostic value, contrast enhancement of the nerve might be able to predict poor outcomes. Facial nerve edema and inflammation increase the severity of facial nerve paralysis, so the increase in contrast enhancement shown on MRI may be correlated with the severity of facial paralysis [7-9]. However, the benefit of contrast-enhanced MRI in determining the prognosis of facial nerve palsy remains controversial.

We describe a case of a 70-year-old Japanese man who developed idiopathic bilateral facial nerve palsy and was able to achieve complete recovery. We demonstrate the usefulness of contrast-enhanced MRI by providing information about localization and the severity of degeneration of facial nerve. Few cases have been reported, so this case report with a literature review may serve as an important discussion point regarding facial nerve palsy.

## Case presentation

**Figure 1:**
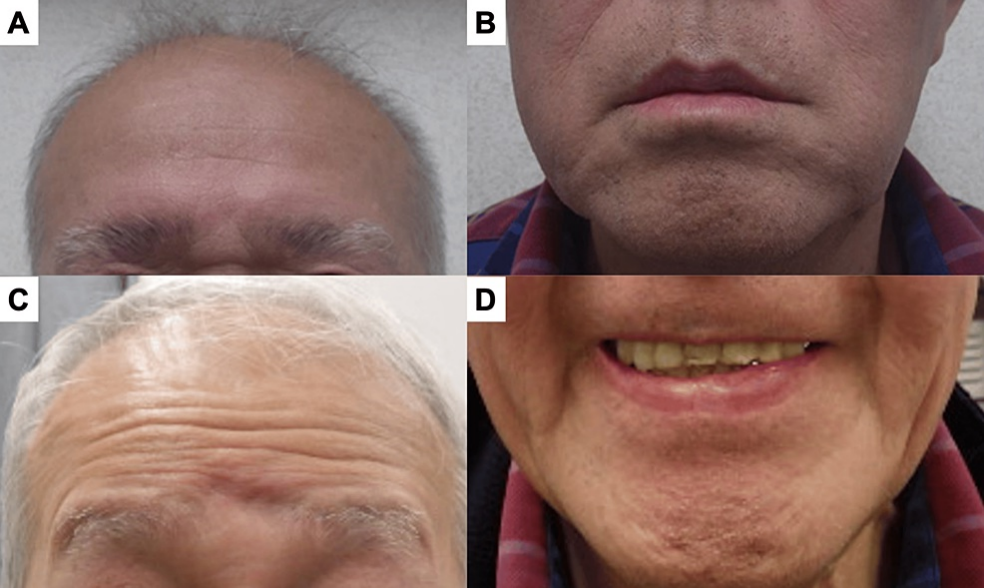
The movements of the forehead and corners of the mouth have improved on both sides when compared before and after treatment A. Forehead at the first visit, B. Corners of mouth at the first visit, C. Forehead on day 240 after onset, D. Corners of mouth on day 240 after onset.

A 70-year-old Japanese man developed complete bilateral facial palsy with Grade VI in the House-Brackmann (HB) grading system and 6 points in the Yanagihara grading system. Oral prednisolone at 45 mg/day was administrated on day 3 after onset. Even on day 7 after onset, he still had complete bilateral facial paralysis with the same grade/score on both scales. He was unable to wrinkle his forehead (Figure 1A) or lift the corners of his mouth (Figure 1B).

He showed no other symptoms, such as fever, chills, myalgia, or headache. There was no history of trauma or visits to an endemic area of Lyme borreliosis in Japan or overseas. He did not show any signs of tick bites, vesicles, lip edema, erythema, or lymphadenopathy, which might have suggested Melkersson-Rosenthal syndrome or Lyme borreliosis.

**Figure 2:**
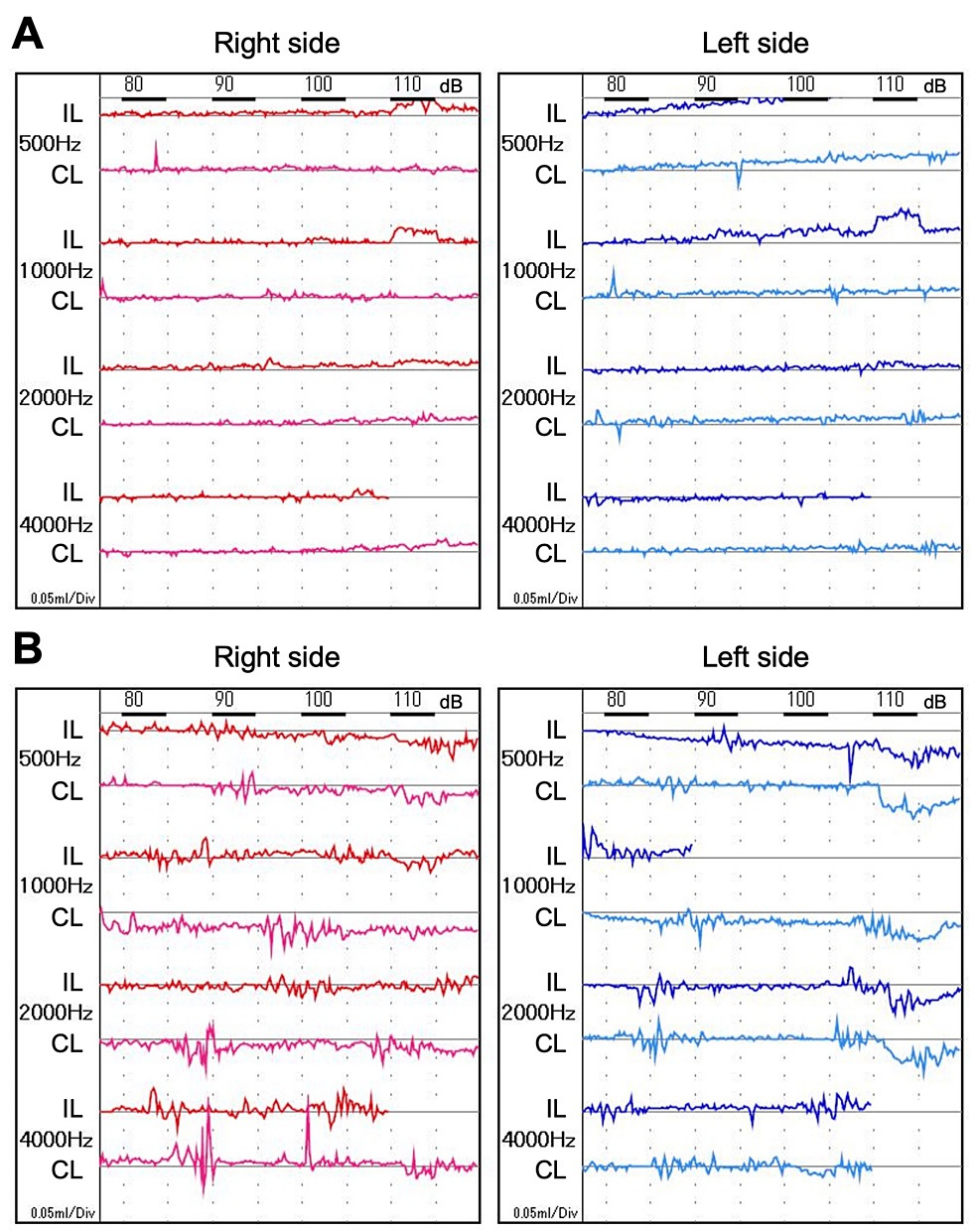
Bilateral stapedial reflexes, which had disappeared before treatment, recovered after treatment A. At the first visit, B. On day 240 after onset. IL; ipsilateral, CL; contralateral.

Acoustic reflexes were absent in the right ear and responded as upward reflex curves in the left ear (Figure 2A).

Schirmer’s test showed decreased tear secretion on the left side (R/L=17/6 mm). Electrogustometry was unremarkable. Serological examination revealed a history of varicella-zoster virus infection, but no evidence of herpes simplex virus-1 or human immunodeficiency virus infection. There were no findings related to anti-neutrophil cytoplasmic antibody-associated vasculitis, sarcoidosis, or malignancy including malignant lymphoma (Table 1).

**Table 1:** Laboratory findings at the first visit WBC: white blood cells, Hb: hemoglobin, PLT: platelets, CRP: C-reactive protein, HSV: herpes simplex virus, VZV: varicella-zoster virus, ACE: angiotensin-converting enzyme inhibitor, HIV-Ab: human immunodeficiency virus antibody, PR3-ANCA: Proteinase 3 anti-neutrophil cytoplasmic antibody, MPO-ANCA: Myeloperoxidase anti-neutrophil cytoplasmic antibody, sIL-2R: soluble interleukin-2 receptor, PMN: polymorphonuclear

Test results for blood and cerebrospinal fluid		
Blood		
Parameters	Results	Normal Range
WBC	10,640 /µl	3,300-8,600 /µl
Hb	16.1 g/dl	13.7-16.8 g/dl
PLT	25.7 x 10^4^ /µl	15.8 x 10^4^-34.8 x 10^4^ /µl
CRP	0.13 mg/dl	≤ 0.14 mg/dl
ACE	14.3 U/l	7-25 U/l
sIL-2R	388 U/ml	122-496 U/ml
Anti-DNA antibody	≤1.7 IU/ml	≤ 40 IU/ml
HSV IgM (EIA) titer	0.05	< 0.8
HSV IgG (EIA) titer	0.4	< 2.0
VZV IgM (EIA) titer	0.07 (< 0.8)	< 0.8
VZV IgG (EIA) titer	38.8 (< 2.0)	< 2.0
HIV-Ab	0.1 S/CO	< 1.0 S/CO
PR3-ANCA	<0.5 IU/ml	< 3.5 IU/ml
MPO-ANCA	<0.5 IU/ml	< 3.5 IU/ml
Cerebrospinal fluid		
Parameters	Results	Normal Range
Color tone	Colorless and transparent	-
Total protein	18 mg/dl	15-45 mg/dl
Glucose	78 mg/dl	50-80 mg/dl
Mononuclear cell	19 /mm^3^	≤ 5 /mm^3^
PMN cell	0 /mm^3^	≤ 5/ mm^3^

Cerebrospinal fluid showed no elevated protein levels characterizing Guillain-Barré syndrome, although the mononuclear cell count was increased to 19 cells/µl (Table 1). Chest radiography showed no hilar lymphadenopathy characteristic of sarcoidosis. Computed tomography showed no temporal bone fracture, middle or inner ear abnormalities, or widening enlargement of the inner ear canal. Plain MRI showed no abnormalities in the brain.

**Figure 3:**
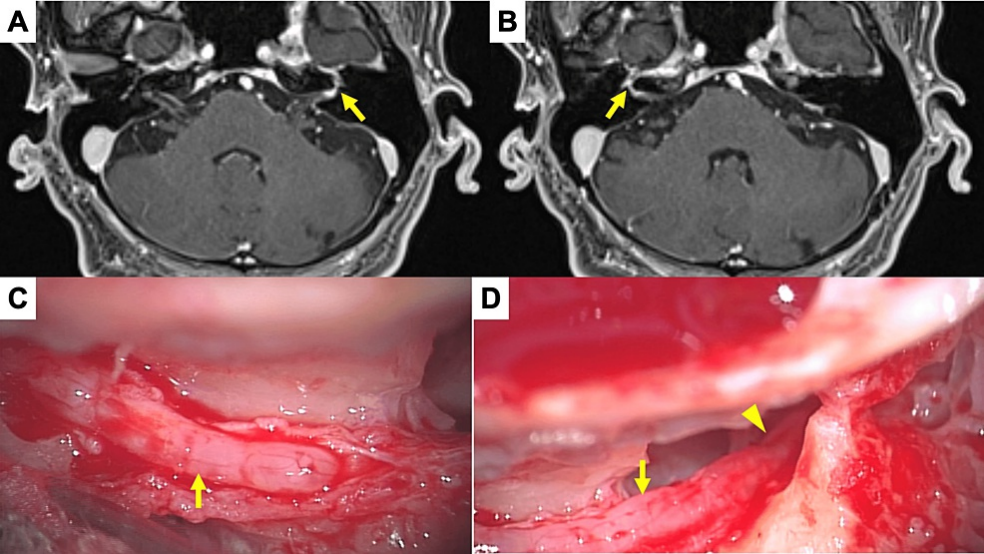
Contrast-enhanced MRI revealed enhancements of bilateral facial nerves with higher signal intensity on the left side and significant edema of facial nerve was observed during surgery A. An axial image of the labyrinthine and geniculate portion of the left facial nerve. The arrow indicates enhanced area. B. An axial image of the labyrinthine and geniculate portion of the right facial nerve. The arrow indicates enhanced area. C. Findings of vertical portion of the left facial nerve after decompression. The arrow indicates the edema of the nerve after cutting the sheath. D. Findings of horizontal-second geniculate portion of the left facial nerve after decompression. The arrow and the arrow head indicate the edematous nerve of second geniculate portion and horizontal portion after cutting the sheath, respectively.

Contrast-enhanced MRI performed on day 7 after onset showed enhanced intensity of bilateral labyrinthine and tympanic segments of the facial nerve, including the geniculate ganglions. There were no other enhanced lesions including in the meningeal, periventricular, supratentorial, or infratentorial regions. The signal intensity on the geniculate ganglion of the left side (Figure 3A) was higher than that on the right side (Figure 3B).

The ratio of the highest signal intensity value on contrast-enhanced T1-weighted images was 1:1.18 (R:L) from the labyrinthine segment to the geniculate ganglion and 1:1.49 (R:L) in the horizontal segment.

The patient was treated with prednisolone tapered from 60 mg/day and 1000 mg/day of valaciclovir for seven days. Bilateral paralysis remained severe 10 days after onset. An electrophysiological test on day 16 after onset showed that the degree of bilateral facial nerve palsy remained unchanged at Grade V on the HB grading system. The NET threshold was 11 mA/20 mA (R/L), although it was difficult to measure compound muscle action potentials bilaterally. On day 32 after onset, the patient had bilateral paralysis with an HB grading system score of II/III (R/L) and Yanagihara grading system score of 34/26 (R/L), and transmastoidal facial nerve decompression was performed on the left side based on the higher signal intensity on contrast-enhanced MRI, the left-right difference in addition to the finding of relative reduction by NET, the reduction of tear secretion on the left side by Schirmer’s test and the inverted responses by acoustic reflex. The facial nerve showed severe edema from the horizontal segment (Figure 3C) to the mastoid segment (Figure 3D) after exposure of the nerve by cutting the sheath. The ossicular chain was preserved during the surgery.

**Figure 4:**
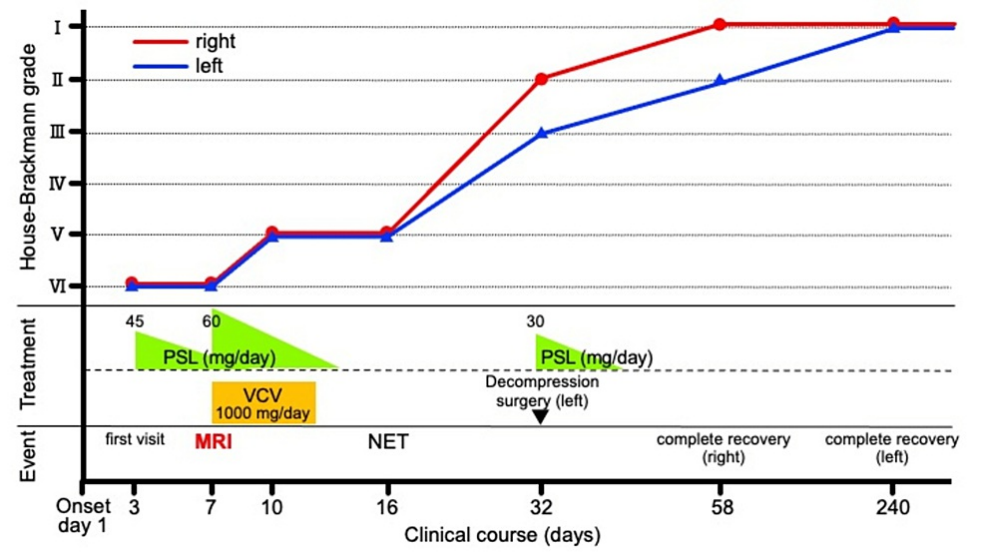
The palsy on the right side showed rapid improvement from day 16 after the onset and healed on day 58, while that on the left side showed slow improvement and took 240 days until the complete recovery PSL: prednisolone, VCV: valacyclovir, NET: nerve excitability test.

On day 240 after onset, the left palsy had completely recovered without hearing loss or any other complications of surgery. He was finally able to wrinkle his forehead (Figure 1C) and lift the corners of his mouth (Figure 1D). Both sides of the acoustic reflex turned positive (Figure 2B). The right-side palsy also fully recovered by day 58 after onset. His clinical course is summarized in Figure 4.

There were no sequelae of fascial palsy including synkinesis, contracture, facial spasms, recurrence of facial palsy, or systemic symptoms for more than 3 years.

## Discussion

The value of MRI in determining the prognosis of idiopathic facial nerve palsy remains controversial. Although electrophysiological tests can predict the prognosis of facial nerve palsy, in the early stages, the results are usually unremarkable. In bilateral cases, there can be difficulty in predicting the prognosis using electrophysiological tests because bilateral nerves are damaged. Contrast-enhanced MRI for facial nerve palsy provides valuable information for evaluating damaged facial nerves [6]. This case report showed that a comparison of signal intensity values of idiopathic bilateral facial nerves by contrast-enhanced MRI may reflect the side of the more severe damage, suggesting the poorer prognosis of paralysis.

A correlation between contrast enhancement and the degree of nerve damage or paralysis has been reported [10,11]. The mechanisms of contrast enhancement by gadolinium may be either hyperemia associated with disruption of the blood-nerve barrier in the perineural structures or endoneural vascular permeability at sites of neural edema and Wallerian degeneration [8,12]. Minakata et al. were the first to quantitatively demonstrate a significant association between increased signal on contrast-enhanced T1-weighted MRI, intracanalicular facial nerve swelling, and improvement in peripheral facial palsy [13]. Kim et al. also reported a better correlation between enhancement on MRI and pathologic changes in the facial nerve, particularly in the labyrinthine segment and geniculate ganglion within three weeks after onset [14].

The prognostic potential of contrast-enhanced MRI has not been adequately discussed, although its diagnostic validity is well documented. We reviewed five reports that addressed the prognostic value of the MRI in facial nerve palsy (Table 2).

**Table 2:** Literature review for the prognostic value of the MRI in facial nerve palsy CMAP: compound muscle action potential, ENoG: electroneurography

Author and reference number [ref no.]	Number of cases (n)	Timing of MRI scan	Protocol for evaluating contrast-enhancement	Clinical and neurophysiological findings	Summary
Engström M, et al. [7]	11	11, 40, and 97 days from the onset (mean)	Scoring according to signal intensity	Yanagihara scale House-Brackmann Grade ENoG	Clinical and neurophysiological improvements in facial nerve function were associated with disappearance of facial nerve enhancement.
Yetiser S, et al. [15]	13	15 days from the onset (mean)	Comparing signal intensity between controls and patients	House-Brackmann Grade	Patients with enhancement took longer to recover.
Kress BP, et al. [16]	20	1st day of inpatient treatment	Signal intensity value	CMAP	Contrast enhancement indicated an unfavorable clinical course and a highly significant pathological CMAP.
Yücel V, et al. [17]	150	9.81 days from the onset (mean)	Contrast-enhanced or not	House-Brackmann Grade	Contrast enhancement showed no significant correlation between pretreatment House–Brackmann grades, clinical recovery and the recurrence rate.
Song MH, et al. [18]	44	4.6 days from the onset (mean)	Comparing of signal intensity between before and after enhancement	House-Brackmann Grade	Signal intensity increase showed no significant difference between different prognostic groups.

Yetiser et al. demonstrated a strong correlation between facial nerve enhancement and the time to recovery. The mean time from the onset of facial paralysis to recovery in patients with enhancement was 14 weeks, whereas the mean time to recovery in patients without enhancement was 6 weeks. Enhancement in more than one segment was shown to negatively affect recovery [15]. Kress et al. reported that MRI has prognostic value in the early stages of Bell's palsy. Patients with MRI findings indicating an unfavorable clinical course had a highly significant pathological compound muscle action potential [16]. In contrast, Engström et al. reported that during the serial follow-up period, the enhancement had disappeared [7]. Although clinical and neurophysiological improvements in facial nerve function during recovery from Bell's palsy were associated with the disappearance of facial nerve enhancement, the presence of enhancement on the initial MRI scan was suggested to not necessarily indicate a poor prognosis for recovery [10]. Yücel et al. reported that there was no significant correlation between pre-treatment HB grades and enhancement and between clinical recovery and enhancement [17]. Song et al. reported that quantitative analysis of the facial nerve on both the lesion side and the normal side, which allowed for more accurate measurement of facial nerve enhancement in patients with facial palsy. They showed a statistically significant correlation with the initial severity of facial nerve inflammation, although little prognostic significance was shown [18]. However, all of these reports were based on unilateral cases, not bilateral cases.

Unlike studies in unilateral facial nerve palsy, there has not yet been a proper evaluation of the utility of contrast-enhanced MRI in bilateral facial nerve. In one case of bilateral facial nerve palsy with left palsy 3 days after right palsy, a predominant intensity enhancement was found in the right facial nerve [19]. In contrast, a case of bilateral facial nerve palsy with right palsy 2 weeks after left palsy showed enhancement of equal intensity on both sides [20]. Our patient had a simultaneous onset of idiopathic bilateral facial palsy and contrast-enhanced MRI was performed one week after the onset. Labyrinthine segments to the geniculate ganglions and the horizontal portion of the left facial nerve had higher contrast than on the right side. The difference in recovery time also suggested that the denervation was more severe on the left than on the right side. Higher MRI signal intensity corresponded with higher NET values and prolonged left palsy, suggesting that contrast-enhanced MRI may help predict prognosis at an early stage. On the other hand, it is considered to be effective only in relative comparisons between left and right within the same individual as the signal intensity by the enhancement may differ among patients and the testing equipment settings and manufacturer. Establishing the predictive value of contrast-enhanced MRI in predicting prognosis is an issue for future consideration.

There are some limitations in the current report. First, this study is based on a single case due to the rarity of the disease. There is a need to accumulate evidence regarding the relationship between the contrast enhancement effect of MRI and the severity and prognosis of bilateral facial nerve palsy. Second, if the damage to the nerves is of the same degree, comparisons between sides become difficult. It is necessary to decide the indication of further treatment based on the course of paralysis and findings such as acoustic reflex and NET.

## Conclusions

This case demonstrates the usefulness of contrast-enhanced MRI as a prognostic predictor of bilateral simultaneous facial nerve palsy, of which prognosis on either side has not yet been established. Comparison of enhancement effects of both sides of facial nerves contributes to estimating the side of more damage, providing information about the further treatment indication after steroid administration such as surgery. In this case, the course on the side with higher contrast enhancement was poorer than that on the other side, and facial nerve decompression was successful in improving the prognosis. The efficacy of contrast-enhanced MRI needs to be clarified by accumulating evidence on idiopathic bilateral facial nerve palsy.
